# On the function of TRAP substrate-binding proteins: the isethionate-specific binding protein IseP

**DOI:** 10.1042/BCJ20240540

**Published:** 2024-12-09

**Authors:** Michael C. Newton-Vesty, Michael J. Currie, James S. Davies, Santosh Panjikar, Ashish Sethi, Andrew E. Whitten, Zachary D. Tillett, David M. Wood, Joshua D. Wright, Michael J. Love, Timothy M. Allison, Sam A. Jamieson, Peter D. Mace, Rachel A. North, Renwick C.J. Dobson

**Affiliations:** 1 Biomolecular Interaction Centre, School of Biological Sciences, MacDiarmid Institute for Advanced Materials and Nanotechnology, University of Canterbury, Christchurch 8140, New Zealand; 2 Australian Research Council Centre for Cryo-electron Microscopy of Membrane Proteins, Bio21 Molecular Science and Biotechnology Institute, University of Melbourne, Parkville, Victoria, Australia; 3 Australian Synchrotron, Australian Nuclear Science and Technology Organisation (ANSTO), 800 Blackburn Road, Clayton, Victoria 3168, Australia; 4 Department of Biochemistry and Molecular Biology, Monash University, Melbourne, Victoria 3800, Australia; 5 Australian Centre for Neutron Scattering (ACNS), ANSTO, Lucas Heights, New South Wales 2234, Australia; 6 Biomolecular Interaction Centre, School of Physical and Chemical Sciences, University of Canterbury, Christchurch 8140, New Zealand; 7 Biochemistry Department, School of Biomedical Sciences, University of Otago, Dunedin 9054, New Zealand; 8 School of Medical Sciences, Faculty of Medicine and Health, University of Sydney, Sydney, New South Wales 2006, Australia; 9 Department of Biochemistry and Pharmacology, Bio21 Molecular Science and Biotechnology Institute, University of Melbourne, Parkville, Victoria 3010, Australia

**Keywords:** *bacterial sulfur metabolism*, *Desulfovibrio*, *isethionate*, *Oleidesulfovibrio alaskensis*, *substrate-binding proteins*, *tripartite ATP-independent periplasmic trap) transporters*

## Abstract

Bacteria evolve mechanisms to compete for limited resources and survive in new niches. Here we study the mechanism of isethionate import from the sulfate-reducing bacterium *Oleidesulfovibrio alaskensis*. The catabolism of isethionate by *Desulfovibrio* species has been implicated in human disease, due to hydrogen sulfide production, and has potential for industrial applications. *O. alaskensis* employs a tripartite ATP-independent periplasmic (TRAP) transporter (*Oa*IsePQM) to import isethionate, which relies on the substrate-binding protein (*Oa*IseP) to scavenge isethionate and deliver it to the membrane transporter component (*Oa*IseQM) for import into the cell. We determined the binding affinity of isethionate to *Oa*IseP by isothermal titration calorimetry, *K*_D_ = 0.95 µM (68% CI = 0.6–1.4 µM), which is weaker compared with other TRAP substrate-binding proteins. The X-ray crystal structures of *Oa*IseP in the ligand-free and isethionate-bound forms were obtained and showed that in the presence of isethionate, *Oa*IseP adopts a closed conformation whereby two domains of the protein fold over the substrate. We serendipitously discovered two crystal forms with sulfonate-containing buffers (HEPES and MES) bound in the isethionate-binding site. However, these do not evoke domain closure, presumably because of the larger ligand size. Together, our data elucidate the molecular details of how a TRAP substrate-binding protein binds a sulfonate-containing substrate, rather than a typical carboxylate-containing substrate. These results may inform future antibiotic development to target TRAP transporters and provide insights into protein engineering of TRAP transporter substrate-binding proteins.

## Introduction

To survive in new niches, bacteria evolve mechanisms to compete for and import resources into the cell. Tripartite ATP-independent periplasmic (TRAP) transporters are secondary transporters that move scavenged organic acids across the plasma membrane of bacteria and archaea by co-transporting sodium ions down an electrochemical gradient [1–4]. Like the ATP-binding cassette importers, TRAP transporters utilise a high-affinity substrate-binding protein (P-subunit) that binds the organic acid and delivers it to the membrane-spanning transporter subunits (Q and M) for uptake [5–7].

**Figure 1.**
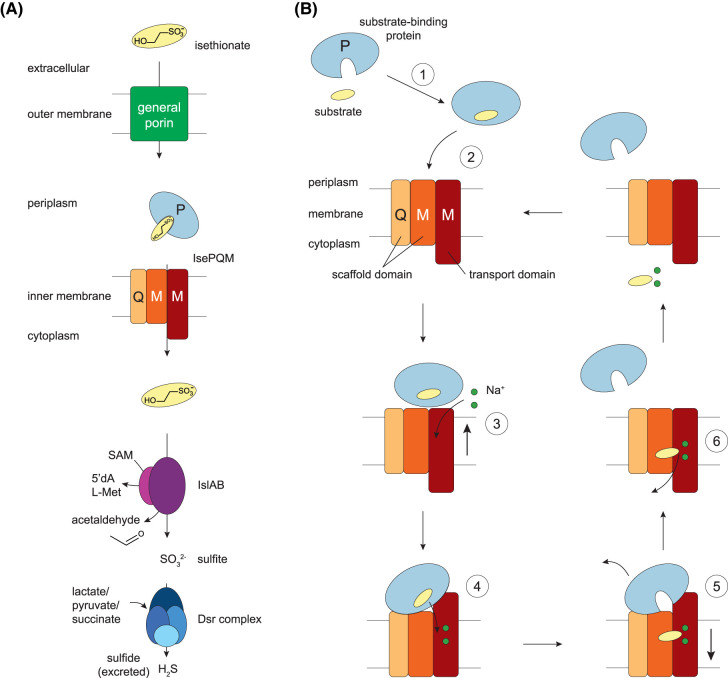
Isethionate metabolism in *Desulfovibrio* species and proposed TRAP transporter mechanism. (**A**) Isethionate catabolism in Gram-negative *O. alaskensis*. Isethionate diffuses into the periplasmic space via a general porin before being transported across the inner membrane by the IsePQM TRAP transporter. In the cytosol, isethionate is broken down into acetaldehyde and sulfite by the isethionate sulfite lyase, IslAB [coupled to the conversion of *S*-adenosylmethionine (SAM), to 5′-deoxyadenosyl-l-methionine (5′dA L-Met)]. The acetaldehyde can be further utilised as a carbon source, or broken down to yield ATP and acetate, which is excreted. The sulfite ion is used as the terminal electron acceptor by the dissimilatory sulfite reductase (Dsr) complex before the final sulfide product is excreted. (**B**) Proposed TRAP transporter cycle, noting that the precise order of events is yet to be determined. (1) The TRAP substrate-binding protein (P) binds the substrate and (2) delivers it to the membrane-spanning domains. (3) The transport domain moves upwards against the scaffold domain, allowing Na^+^ ions to bind the transport domain. (4) The substrate-binding protein opens to release the substrate to a pocket in the transport domain. (5) The substrate-binding protein diffuses away, and the transport domain moves downward, (6) releasing the substrate and Na^+^ ions to the cytoplasm.

*Oleidesulfovibrio alaskensis* G20 (formerly annotated as *Desulfovibrio alaskensis*) is a Gram-negative, moderately halophilic, mesophilic, and strict anaerobe that was isolated from an oil well in Alaska [8]. It is a sulfate-reducing bacterium that reduces various oxidised organic sulfur compounds (Figure 1A), which function as the terminal electron acceptors in the process of anaerobic respiration. Specifically, it can reduce sulfate, sulfite, and thiosulfate to produce sulfide, whilst utilising lactate, pyruvate, and succinate as electron donors [8,9]. Production of hydrogen sulfide by *Desulfovibrio* species in the human gut has been implicated in a range of human diseases including ulcerative colitis, irritable bowel syndrome and colorectal cancer [10–12]. Moreover, *Desulfovibrio* prove problematic in industrial applications, with the production of sulfides contributing to the corrosion of equipment [13]. The potential of *Desulfovibrio* for bioremediation of sulfates in wastewater is also currently being explored as a viable solution to contamination [14].

While investigating organosulfonate metabolism in *O. alaskensis*, Peck et al. [15] identified a putative TRAP transporter responsible for 2-hydroxyethane sulfonate (isethionate) import (referred to as DctPQM in their work, hereby referred to as IsePQM). Knockout studies demonstrated that disruption of the identified *iseQM* or the *iseP* genes, abolished growth on isethionate, suggesting that the encoded IsePQM TRAP system is indeed an isethionate transporter. Peck et al. [15] also identified gene clusters that include *isePQM* genes in other *Desulfovibrio* strains, indicating that TRAP transporters are generally utilised by *Desulfovibrio* species. Using differential scanning fluorimetry (DSF), they estimated the dissociation constant (*K*_D_) for isethionate to recombinant IseP from *O. alaskensis* (*Oa*IseP) to be 1.7 ± 0.1 mM, while analogues of isethionate (taurine, hypotaurine, sulfoacetaldehyde, and ethylsulfonate) did not show significant binding [15]. Unlike other TRAP substrate-binding proteins, the reported *K*_D_ of *Oa*IseP for isethionate was unusually low (most are in the nM range), which could be a feature of the method used to determine binding.

Recent structural studies of the sialic acid TRAP transporters have extended our understanding of transport at the molecular level [16–23]. In the sialic acid-specific TRAP system (SiaPQM), the soluble substrate-binding protein SiaP is required for transport [24] and associates with the plasma membrane-spanning SiaQM transporter component to facilitate the translocation of sialic acid across the membrane (Figure 1B). These studies suggest that TRAP transporters use an elevator-type mechanism. However, the conformational coupling between the soluble substrate-binding protein and the SiaQM membrane transporter is not fully understood. Currently, there are 23 high-resolution structures available for sialic acid-specific SiaP (and various mutants), largely from five species of Gram-negative bacteria: *Haemophilus influenzae* [17,22,25–27], *Vibrio cholerae* [16,18,28], *Photobacterium profundum* [20], *Fusobacterium nucleatum* [28] and *Pasteurella multocida* [28]. As such, we have a good understanding of sialic acid substrate-binding proteins and the molecular basis for their high affinity and specificity. However, much less is known about TRAP substrate-binding proteins that bind other organic acids.

In a structural biology tour de force, Vetting et al. [6] developed a high-throughput protein production and DSF pipeline to investigate TRAP transporter substrate-binding proteins. The study highlighted the potential of using mass spectrometry and crystallographic approaches with entire metabolomes as screening libraries. They identified 40 novel substrates through these methods and determined 60 crystal structures (representing 46 unique proteins), with 51 containing bound ligands. Currently, there are ∼70 crystal structures for unique substrate-binding proteins of TRAP transporters deposited into the Protein Data Bank (PDB) with ∼110 total deposited structures.

The structure of TRAP substrate-binding proteins comprises two globular lobe-like domains connected by a variable hinge region [29], which is formed by two β-strands and a unique extended α-helix. Substrates bind within the cleft between the two domains, which close around the bound substrate via a hinge-bending conformational change often described as a ‘Venus flytrap’ mechanism [30,31]. The structures for ligand-free SiaP adopt open conformations, suggesting that closure is strictly substrate-induced, consistent with a two-state induced fit binding model [25,32]. Conformational dynamics have been investigated with pulsed electron-electron double resonance (PELDOR) spectroscopy and single-molecule Förster Resonance Energy Transfer (smFRET), which support the proposal that *V. cholerae* SiaP (*Vc*SiaP) exclusively occupies an open conformation in the absence of substrate and that substrate binding induces and stabilises closure [16,18]. While these experiments provide excellent data on the rates of opening and closure, a recent study describes discrepancies between measurements for *H. influenzae* SiaP (*Hi*SiaP) obtained via PELDOR and smFRET due to technical artefacts [18]. They found *Hi*SiaP adopted a more closed conformation in solution, making it unclear whether this model can be applied to all TRAP substrate-binding proteins, or whether there is variation between isozymes. Additionally, recent research on *Aggregatibacter actinomycetemcomitans* SiaP (*Aa*SiaP) found a non-cognate ligand, acetate, stabilised a partially closed conformation [32]. Molecular dynamics simulations indicated that the open-unliganded form of *Aa*SiaP can sample the closed conformation, further complicating the substrate binding mechanism of SiaP.

Here, we report the functional, biophysical, and structural characterisation of the isethionate substrate-binding protein of the TRAP transporter from *O. alaskensis* (*Oa*IseP) to elucidate the molecular details of how a TRAP substrate-binding protein binds sulfonate-containing substrates, rather than the typical carboxylate-containing substrates.

## Results

### Bioinformatic analysis demonstrates that *Oa*IseP shares key residues for isethionate binding

**Figure 2.**
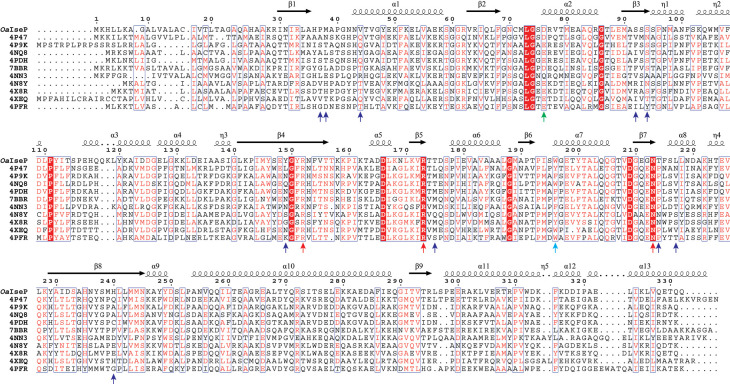
Multiple sequence alignment of TRAP substrate-binding proteins homologous to *O. alaskensis* IseP (*Oa*IseP), with structures deposited in the PDB. Residue numbering and secondary structure are according to the structure of the sequence with the highest identity (34%), a substrate-binding protein from *Brucella anthropi* (PDB ID: 4P47). Residues highlighted in red indicate complete sequence identity, while red letters indicate residues with similar physicochemical properties and blue boxes denote similarity between groups. Conserved arginine and asparagine substrate-binding residues are marked with red arrows, conserved aromatic residues are marked with a cyan arrow, conserved aspartate and glutamate residues are marked with a green arrow and other variable binding site residues are marked with blue arrows. Created in ESPript 3.0, https://espript.ibcp.fr [49].

A bioinformatic search for homologues to *Oa*IseP with known structures was carried out to glean insights into the mechanism of sulfonate-containing substrate binding. We performed a search using the Position-Specific Iterated Basic Local Alignment Search Tool (PSI-BLAST) [33] and obtained 10 sequences of homologous proteins (29–34% sequence identity) with known crystal structures (Figure 2).

Bacterial species from a range of environments were represented including those that commonly inhabit soil [34], groundwater [35] and compost [36], as well as some pathogenic to humans [37] and small mammals [37,38]. All the bacterial species were also Gram-negative (Supplementary Table S1). Interestingly, two sulfate-reducing *Desulfovibrio* species had matched homologues [*Maridesulfovibrio salexigens* DSM 2638 (PDB ID: 4NN3) and *Nitratidesulfovibrio vulgaris* RCH1 (PDB ID: 4XEQ)], but no substrate-binding proteins bound to phosphate-containing substrates were identified in the search. While sulfonate- or phosphate-containing substrates share a similar tetrahedral arrangement of the oxygen atoms around the sulfur or phosphorus centres, suggesting a similar binding mode to *Oa*IseP, the lack of sequence similarity suggests that *Oa*IseP may have a different substrate-binding mode. There were three sugar-binding homologues (PDB ID: 7BBR, bound to 2-keto-3-deoxy-d-gluconate; 4N8Y, bound to galacturonate and 4X8R bound to glucuronate), but none of the most widely studied sialic acid-specific substrate-binding proteins.

Two were structures in an open conformation (PDB IDs: 4P47 and 4PFR) and the rest were in the closed conformation bound to pantoate or erythronate (Supplementary Figure S1A). The structures that contained bound ligands were surveyed to identify key substrate-binding residues (Figure 2). However, most crystallised ligands were co-purified and may not be the native substrates of the TRAP transporter system [6].

Among the sequences obtained in the analysis, the typical substrate carboxylate-binding residues are almost completely conserved [Arg152, Arg173 and Asn213 (4P47 numbering)] (Figure 2). In all the corresponding structures Arg173 forms a salt bridge with the carboxylate group of the ligand, while Arg152 and Asn213 provide supporting hydrogen bonds (Supplementary Figure S1B). These residues are conserved in the sequence of *Oa*IseP, as well as the aromatic residue at position 196, which closes the binding site and provides a hydrophobic face for the carbon backbone of the ligands. An acidic residue is conserved at position 76, aside from an asparagine residue in the 4NN3 structure. This acidic residue typically forms a hydrogen bond with a ligand hydroxyl group. The rest of the binding sites shared some common features in places but were largely variable. Interestingly, the 4NN3 structure was bound to orotic acid, which does not present an additional hydroxyl group in the vicinity of the carboxylate for the conserved arginine and asparagine residues to bind. Thus, the other structures all contain the two conserved arginine residues, but 4NN3 has a lysine directed away from the ligand at position 152 instead (Supplementary Figure S1C).

Overall, comparison of closely related structures with other bound substrates provides useful information for assessing a general mechanism of substrate binding.

### Recombinant *Oa*IseP binds isethionate

Next, we overexpressed the *O. alaskensis iseP* gene in *Escherichia coli* BL21 (DE3) and purified recombinant *Oa*IseP for structural and biophysical analyses (protein sequences used are reported in Supplementary Table S2). *Oa*IseP eluted from the size-exclusion chromatography column as a single symmetrical peak at ∼95 ml and was highly purified based on SDS–PAGE analysis (Supplementary Figure S2A,B). This suggested that *Oa*IseP is a monodisperse species that remains stable throughout purification. We measured the protein mass by native mass spectrometry, which indicated *Oa*IseP is a monomer with a mass of 35 202 ± 6 Da compared with an expected monomeric mass of 35 204 Da based on the protein sequence (Supplementary Figure S2C).

**Figure 3.**
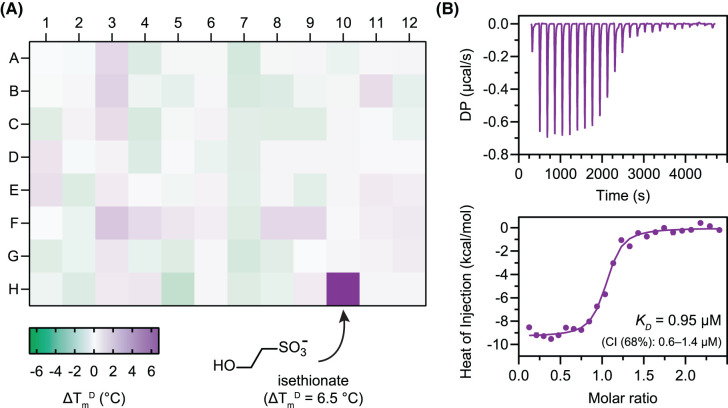
*Oa*IseP substrate binding studies. (**A**) Thermal shift heatmap for *Oa*IseP displaying changes in melting temperature ($\Delta T_{m}^{D}$, °C) with various phosphate and sulfur metabolites. The mean from technical quadruplicates of each condition is shown. Isethionate (position H10) was the only hit ($\Delta T_{m}^{D}$ > 2°C) amongst 94 metabolites from the Phenotype MicroArray PM4A screen. These data are also tabulated in Supplementary Table S3. (**B**) *Oa*IseP binding curve and affinity (*K_D_*) for isethionate determined by ITC. This experiment was conducted with technical triplicates and a representative result is shown. When error bars are not apparent, they are smaller than the data points. These data are also tabulated in Table 1.

To evaluate the substrate specificity of *Oa*IseP, we used DSF to screen a Phenotype MicroArray PM4A Biolog Microplate, which includes isethionate as well as a broader set of phosphate- and sulfur-containing compounds. Across the 94 compounds, only isethionate showed a significant difference in melting temperature, with a 6.52 ± 0.18°C shift (Figure 3A, Table 1 and Supplementary Table S3). This finding is consistent with substrate-binding proteins in general, which are highly specific and have a high affinity for their substrate [2].

**Table 1.** Thermal stability and isothermal titration calorimetry for isethionate binding

**Thermal stability (** $T_{m}^{D}$ **, °C) ± SD from DSF**	
*Oa*IseP	**57.9 ** **±** ** 0.2**
*Oa*IseP PM4A phosphate control well (A1)	**57.4 ** **±** ** 0.2**
*Oa*IseP PM4A sulfur control well (F1)	**57.2 ** **±** ** 0.2**
*Oa*IseP** **+** **isethionate (PM4A, well H10)	**63.7 ** **±** ** 0.2**
*Oa*IseP** **+** **isethionate (0.1 mM)	**64.0 ** **±** ** 0.2**
*Oa*IseP** **+** **isethionate (5 mM)	**74.0 ** **±** ** 0.2**
*Oa*IseP** **+** **MES (5 mM)	**58.5 ** **±** ** 0.3**
*Oa*IseP** **+** **HEPES (5 mM)	**57.0 ** **±** ** 0.6**
**Binding affinity from ITC**	
*K*_D_ isethionate (68% CI) µM	**0.95 (0.6–1.4)**
*n* (fixed)	**1**
Δ*H* (kcal/mol)	**−9.5 (−9.1 to −9.8)**
Δ*S* (cal/mol)	**−4.2**
Δ*G* (kcal/mol)	**−8** **.** **2**

Thermal stability of *Oa*IseP binding isethionate using DSF, where the thermal stability is reported as the first derivative ($T_{m}^{D}$) and the error is the standard deviation of quadruplicate measurements. Dissociation constant (*K*_D_) values were determined for *Oa*IseP binding isethionate using ITC, where the values in brackets refer to the 68% confidence interval.

To further characterise isethionate binding, DSF thermal shift assays with *Oa*IseP were conducted with isethionate prepared in-house and compared with the results obtained from the Biolog screen (Table 1). The melting temperature of *Oa*IseP ($T_{m}^{D}$) was 57.9 ± 0.2°C in buffer (50 mM Tris pH 8.0, 150 mM NaCl) and was stabilised in the presence of isethionate ($T_{m}^{D}$ with 0.1 mM isethionate is 64.0 ± 0.2°C [+6.1°C] and with 5 mM is 74.0 ± 0.2°C [+16.1°C]). Isothermal titration calorimetry (ITC) was used to measure the dissociation constant (*K*_D_) and thermodynamic parameters of the interactions between *Oa*IseP and the substrate isethionate (Figure 3B). The *K*_D_ for this interaction is 0.95 µM (68% confidence interval = 0.6–1.4 µM), which is a weaker affinity compared with published values for other TRAP substrate-binding proteins, although it is still in the nanomolar range. For example, the *K*_D_ for sialic acid binding to *F. nucleatum, P. multocida, H. influenzae* and *V. cholerae* SiaP are 0.03–0.30 µM [28]. Other TRAP substrate-binding proteins with different substrates also displayed affinities in the sub-micromolar (nanomolar) range; such as *Bordetella pertussis* DctP7 binding pyroglutamate with a *K*_D_ of 0.3 µM [39], TeaA to ectoine with a *K*_D_ of 0.19 µM [40], and various species’ TarP proteins binding similar substrates with *K*_D_ values of 0.008–0.5 µM [41]. While TRAP substrate-binding proteins are typically considered highly substrate-specific, the CxaP sugar acid substrate-binding protein of *Advenella mimigardefordensis* strain DPN7^T^ has been demonstrated to bind and transport several different sugars with moderate structural differences (*K*_D_ values of 1.9–8.0 µM) [42]. *Oa*IseP was also consistent with other TRAP substrate-binding proteins, with the interaction largely enthalpically driven (Δ*H* = −9.5 kcal/mol) and a small negative entropic contribution (Δ*S* = −4.2 cal/mol). This suggests that the interaction is largely driven by the formation of new hydrogen bonds, van der Waals interactions and the conserved arginine salt bridge typical of the family.

Overall, DSF and ITC assays demonstrate that *Oa*IseP is functional in that it specifically binds isethionate in the high nanomolar range.

### The structure of ligand-free *Oa*IseP

We first determined the crystal structure of ligand-free *Oa*IseP. TRAP substrate-binding proteins are generally very stable (as is *Oa*IseP, thermal stability shown in Table 1) and crystallise readily. We found that *Oa*IseP crystallised in more than 50 conditions from just three 96-condition commercial screens, of which we screened 10 of these ‘hits’ for data quality. From these, three high-resolution datasets were obtained to 1.48, 1.65 and 1.89 Å. Data collection statistics are reported in Table 2. Of these three datasets, only the 1.48 Å dataset was truly ligand-free, whereas the other two datasets had sulfonate-containing buffering compounds [4-(2-hydroxyethyl)-1-piperazineethanesulfonic acid (HEPES) and 2-(*N*-morpholino)ethanesulfonic acid (MES)] in the substrate-binding site arising from the crystallisation condition (see crystallisation and structure determination methods). For the truly ligand-free structure, we solved the initial phases using molecular replacement with *Hi*SiaP (PDB ID: 2CEY). From the initial phases, the electron density could be well modelled (except for the first four residues of the native sequence) and resulted in a structure with an *R*_free_ of 0.211, no Ramachandran outliers, and good geometry (Table 2).

**Table 2.** Data collection and refinement statistics for *Oa*IseP

	**Ligand-free**	**Isethionate-bound**	**HEPES-bound**	**MES-bound**
**Data collection**				
Temperature (*K*)	100	100	100	100
Detector	EIGER X 16M	EIGER X 16M	EIGER X 16M	EIGER X 16M
Crystal-to-detector distance (mm)	200	110	300	260
Space group	*P* 2_1_2_1_2_1_	*P* 2_1_2_1_2_1_	*P* 2_1_2_1_2_1_	*P* 2_1_2_1_2_1_
Unit cell parameters [*a*, *b*, *c* (Å)]	42.9, 73.0, 95.2	72.2, 83.2, 91.9	45.6, 73.2, 94.8	43.7, 73.2, 95.5
Resolution range (Å)	47.6–1.48 (1.51–1.48)	46.9–1.25 (1.27–1.25)	47.4–1.89 (1.93–1.89)	47.7–1.65 (1.68–1.65)
No. of unique reflections	50 754 (2453)	150 942 (7262)	26 163 (1647)	37 696 (1862)
Multiplicity	19.8 (20.5)	48.5 (21.3)	26.2 (26.0)	19.1 (13.0)
Completeness (%)	100 (100)	98.7 (96.9)	100 (99.7)	100 (99.9)
*I*/*σ*(*I*)	25.6 (5.7)	23.9 (2.3)	35.7 (8.1)	15.9 (1.5)
*CC*_1/2_	1.00 (0.98)	1.00 (0.76)	1.00 (0.98)	1.00 (0.76)
*R*_merge_	0.067 (0.550)	0.102 (1.65)	0.059 (0.479)	0.088 (1.72)
**Refinement statistics**				
No. of reflections used	50 684	150 942	26 109	37 634
No. of protein atoms (non-hydrogen)	2542	5092	2410	2466
No. of water molecules	393	615	179	171
*R*_work_	0.170	0.146	0.174	0.197
*R*_free_	0.211	0.170	0.229	0.241
Reflections used for *R*_free_	2541	7483	1371	1908
Ramachandran favoured, allowed, disallowed (%)	98, 2, 0	98, 2, 0	98, 2, 0	99, 1, 0
RMS bond lengths (Å)	0.0111	0.0121	0.0087	0.0072
RMS angles (°)	2.018	2.014	1.97	1.52
Overall B-factor (protein, waters, ligand)	22.5, 32.9	18.5, 28.4, 12.0	34.1, 42.1, 36.9	37.8, 41.8, 44.2
Clash score	7.58	3.93	2.69	3.01
PDB ID	8T9T	8TE9	8TRP	8TQN

Values in parentheses correspond to the outer shell.

**Figure 4.**
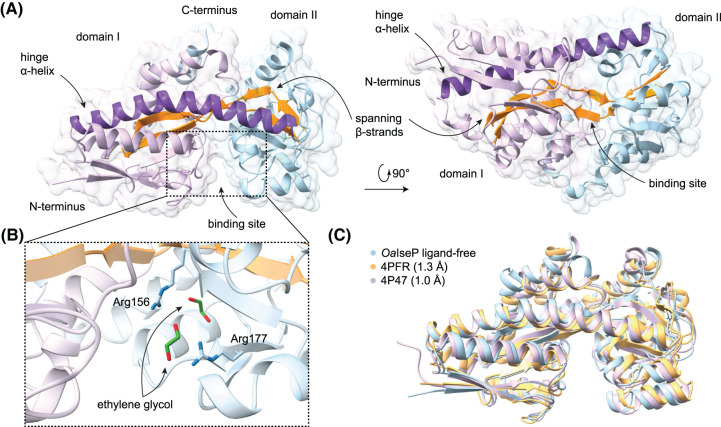
Structure of *Oa*IseP in the ligand-free conformation. (**A**) The overall structure of *Oa*IseP, highlighting the binding site, hinge α-helix (purple) and spanning β-strands (orange). Domain I is coloured lilac, while domain II is coloured light blue. (**B**) Close-up of the binding site of *Oa*IseP, showing the two conserved arginine residues (Arg156 and Arg177) and two molecules of ethylene glycol. (**C**) Structural alignment of *Oa*IseP with two homologous TRAP substrate-binding proteins identified in the bioinformatic analysis, all in the ligand-free conformation (PDB IDs: 4P47 from *Brucella anthropi* and 4PFR from *Cereibacter sphaeroides*).

The structure of ligand-free *Oa*IseP displays the typical TRAP substrate-binding protein architecture (Figure 4A), comprising two domains connected by a spanning pair of disjointed β-strands and a hinging α-helix. The two domains, commonly referred to as domain I (N-terminal domain, residues 1–153 and 243–281) and domain II (C-terminal domain, residues 154–242 and 282–336), are made up of short sets of α-helices and β-strands, with the substrate-binding site residing between them. The two conserved arginine residues (Arg156 and Arg177) that are typically involved with coordinating the carboxylate group of substrates in other TRAP substate-binding proteins (Figure 2), characteristically protrude from domain II (Figure 4B). The first, Arg156, is in proximity to a cryoprotectant molecule of ethylene glycol residing inside domain II, while Arg177 is hydrogen bonding another ethylene glycol molecule. The binding site is filled with water molecules and interestingly, alternate sidechain conformations are present for the Tyr153, Ser220, Leu221 and Ser242 residues lining the binding site, suggesting that binding site residues are dynamic in this conformation.

Of the TRAP substrate-binding protein structures identified in the bioinformatic analysis (Figure 2), two were in the open conformation (PDB IDs: 4P47 and 4PFR). These were aligned with *Oa*IseP (Figure 4C), demonstrating their structural conservation despite different substrate specificities, and confirming while ligand-free, *Oa*IseP is also in the open conformation. The root mean square deviation (r.m.s.d.) of ligand-free *Oa*IseP to 4P47 = 1.0 Å (across 254 α-carbons) and to 4PFR = 1.3 Å (across 178 α-carbons).

### The structure of isethionate-bound *Oa*IseP

To define the molecular details of substrate-binding, we determined the isethionate-bound structure of *Oa*IseP. *Oa*IseP purified in 5 mM isethionate produced crystals that diffracted to 1.25 Å. Using the refined ligand-free structure for molecular replacement, we refined the isethionate-bound structure to an *R*_free_ of 0.170, with no Ramachandran outliers and good geometry (Table 2).

**Figure 5.**
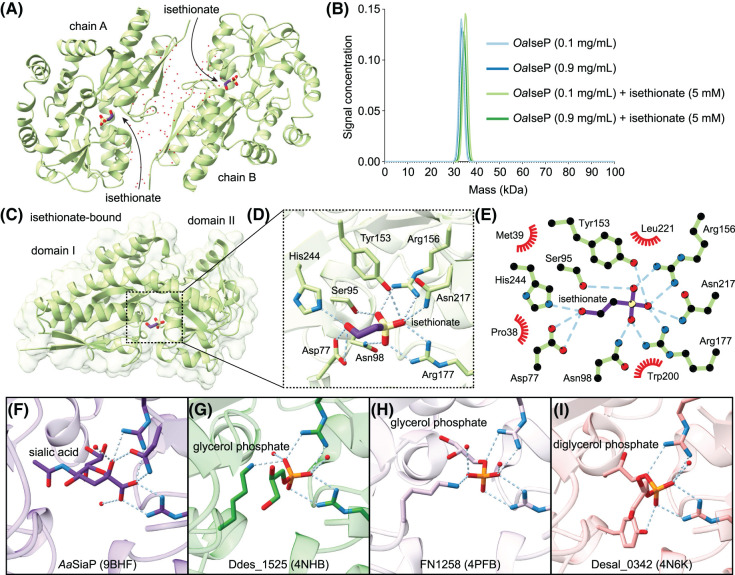
Structure of isethionate-bound *Oa*IseP. (**A**) Asymmetric unit of the isethionate-bound crystal structure of *Oa*IseP containing two monomers. Both monomers are in the closed conformation and bind isethionate identically. (**B**) AUC analysis showing *Oa*IseP is monomeric in solution (∼34 kDa, theoretical molecular mass from amino acid sequence = 35.2 kDa) and the size does not change significantly with the addition of isethionate. (**C**) Cartoon view of *Oa*IseP in the closed conformation, showing isethionate occupying the binding site. (**D**) A focus on the isethionate-binding site showing the hydrogen bonds and salt bridges formed upon isethionate binding. No water molecules are involved in hydrogen bonds with isethionate. (**E**) LigPlot^+^ [66] analysis of isethionate binding, illustrating all the interactions between isethionate and *Oa*IseP. (**F–I**) Carboxylate and phosphate coordination in *Aa*SiaP (PDB ID: 9BFH), Ddes_1525 (PDB ID: 4NHB), FN1258 (PDB ID: 4PFB), and Desal_0342 (PDB ID: 4N6K), highlighting that the phosphate groups of glycerol phosphate are held between the two conserved arginine residues, mimicking the carboxylate of sialic acid, and the different orientations substrates assume in the binding site [6,32]. Only water molecules forming hydrogen bonds with the substrate carboxyl or phosphate groups are shown for clarity.

The asymmetric unit for isethionate-bound *Oa*IseP contained two monomers (Figure 5A) that are bridged by water molecules, where domains I and II of each monomer interact with domains II and I of the opposing monomer. For TRAP substrate-binding proteins, there is only one reported example of a functional dimer forming in solution, with an unusual helix-swapped conformation [43]. Here, the interface of the ‘helix swapped’ dimer is primarily at the C-terminus, as opposed to the binding site as in the isethionate-bound *Oa*IseP structure. We used Proteins, Interfaces, Structures and Assemblies (PISA) to assess the nature of the interface between chains A and B, calculating the size of the buried area to be 624 Å^2^, with a Complexation Significance Score of 0, which suggested that the formation of this assembly was not likely [44]. To verify the monomeric state in solution, we conducted an analytical ultracentrifugation (AUC) sedimentation velocity experiment in the presence and absence of 5 mM isethionate (Figure 5B and Supplementary Figure S3). *Oa*IseP in both conditions displayed a single symmetrical peak at ∼34 kDa, matching the theoretical molecular mass (35.2 kDa) of the monomer calculated from the amino acid sequence.

Isethionate is bound in the cleft between domain I and domain II, with a closer examination of the isethionate-binding site revealing several key contacts made by *Oa*IseP (Figure 5C). The sulfonate moiety of isethionate forms salt bridges with Arg156 and Arg177, as well as forming hydrogen bonds with Ser95, Asn98, Tyr153, and Asn217 (Figure 5D). At the opposing end of isethionate, hydrogen bonds are formed with Asn77 and His244 via the terminal hydroxyl group. No water molecules directly contact isethionate, and the binding site is lined with Pro38, Met39 and Leu221, with Trp200 occupying the bottom of the site (Figure 5E), providing a large hydrophobic surface for the hydrocarbon backbone of isethionate. The structure shows that the sulfonate group of isethionate mimics the three oxygen atoms presented by the carboxylate and hydroxyl groups of the well-studied substrate sialic acid (Figure 5F).

Currently, there are no reported crystal structures of TRAP substrate-binding proteins containing a sulfonate substrate, but there are three structures bound to substrates with a phosphate moiety [Ddes_1525 from *Desulfovibrio desulfuricans* (PDB ID: 4NHB), FN1258 from *F. nucleatum* (PDB ID: 4PFB) and Desal_0342 from *Desulfovibrio salexigens* (PDB ID: 4N6K)] [6]. These occupy various orientations within their respective binding sites compared with isethionate-bound *Oa*IseP, despite the phosphate groups being similarly bound by salt bridges to their Arg156 and Arg177 equivalents. Glycerol phosphate is bound to the proteins Ddes_1525 and FN1258, with the glycerol moiety pointing down towards the domain I and II interface or upwards into α3 of domain I, respectively (Figure 5G,H). Diglycerol phosphate is bound to Desal_0342 with one glycerol moiety pointing down similarly to Ddes_1525, and the second pointing into domain I (Figure 5I). In comparison with *Oa*IseP, which shares an asparagine residue (Asn217) with the sialic acid substrate-binding protein *Aa*SiaP (PDB ID: 9BHF) to co-ordinate their respective sulfonate or carboxylate groups (Figure 5D,F), the two glycerol-phosphate-bound structures lack the equivalent residue. Instead, they rely on a lysine residue from domain I to co-ordinate the phosphate group, and in the diglycerol-phosphate-bound Desal_0342, a tyrosine residue from domain I completes this coordination. In each of these phosphate-bound structures, the equivalent hydrogen bond with Asn217 of *Oa*IseP is replaced with a water molecule positioned between the conserved arginine residues. The glycerol-phosphate-bound structures also have an additional water molecule in separate positions coordinating the phosphate group, which is lacking in the diglycerol-phosphate-bound structure (Figure 5G–I).

### Conformational change upon isethionate binding

**Figure 6.**
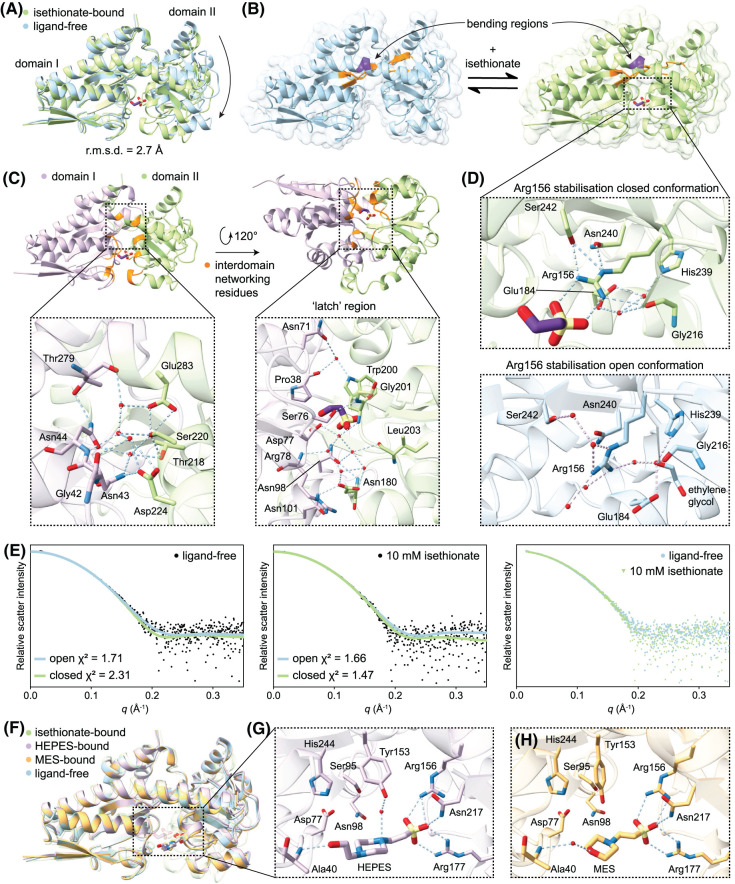
Conformational change of *Oa*IseP upon substrate binding. (**A**) Alignment of isethionate-bound (green) and ligand-free (blue) *Oa*IseP structures, exhibiting the large domain movement to encapsulate isethionate. (**B**) Cartoon views of the *Oa*IseP conformations highlighting the bending regions in the spanning α-helix (purple) and β-strands (orange). (**C**) Overview of the residues (orange) involved in new hydrogen bonding networks between domain I (lilac) and domain II (light green), as well as close-up views of these networks. (**D**) Focus on the coordination of Arg156 in the closed and open *Oa*IseP crystal structures. (**E**) SAXS data for *Oa*IseP in the absence and presence of 10 mM isethionate. Crystal structure theoretical scatter fits using *CRYSOL* are overlayed onto the scatter, with corresponding *χ*^2^ values. (**F**) Alignment of the three ligand-bound structures of *Oa*IseP with the ligand-free structure, demonstrating that only when isethionate is bound does *Oa*IseP fully close. The isethionate-bound structure is transparent to demonstrate the HEPES- and MES-bound structures are in an almost identical conformation to ligand-free *Oa*IseP. (**G**) Focus on the residues involved in binding HEPES. (**H**) Focus on the residues involved in binding MES. Only water molecules that form hydrogen bonds with the ligands have been included.

Next, we compared the *Oa*IseP structures to define the conformational change upon isethionate binding. Aligning the open ligand-free and closed isethionate-bound structures gives an r.m.s.d. of 2.7 Å over all 308 pairs of α-carbons (Figure 6A). The conformational change primarily involves bending of the hinge region within the spanning α-helix and nearby β-strands that connect the two domains. Most notably, a significant bend is introduced at residues 279–281 of this spanning helix, while a broader range of residues are involved in the spanning β-strands at approximately positions 151–157 and 236–244 (Figure 6B). These movements agree with those described in other TRAP substrate-binding proteins, with the spanning α-helix postulated to function as an energetic barrier for domain closure [27].

As well as the direct contacts made to isethionate previously described, *Oa*IseP makes several new contacts bridging the two domains along the binding site interface (Figure 6C). Most notably, hydrogen bonds form between Asn43 (domain I) and Asp224 (domain II), Tyr153 (domain I) and Asn217 (domain II), Ser76 (domain I) and Gly201 (backbone amide, domain II), and Asp110 (domain I) and Ser181 (domain II). Lastly, the sidechains of Asn98 and Asn101 (domain I) both form hydrogen bonds to Asp180 (domain II) in the ‘latch’ region described in other TRAP substrate-binding proteins [20,32]. While no water molecules form hydrogen bonds to isethionate, networks of water molecules further mediate the bridging of the domains upon isethionate binding. A pocket of waters assists with bridging the ‘latch’ region, and a single water molecule co-ordinates the side chain of Asn71 (domain I), the carbonyl backbone of Pro38 (domain I) and Tyr200 (domain II) closing off the bottom of the binding site. In comparison with the ligand-free structure, a distinct water network also forms between the domains at the inner edge of the spanning α-helix (Figure 6C).

In sialic acid substrate-binding proteins SiaP, the ‘latch’ region has been demonstrated to be important for the overall function of the SiaPQM system [20,26]. It consists of a conserved (between SiaP homologues) set of ordered water molecules and residues that form a hydrogen bonding network from the substrate to the bottom face of SiaP, where the interaction with SiaQM likely takes place. In the *Oa*IseP latch region, a similar network exists; but it is slightly disturbed due to the crystal packing conditions. In both chains, Arg78 and Asn101 interact with monomers in neighbouring asymmetric units, making their likely orientation in the native monomeric state unclear. In Figure 6C, chain B of isethionate-bound *Oa*IseP is shown with the alternate sidechain conformations of Arg78 and Asn101 more closely resembling the stacking interactions documented for SiaP. This latch region is likely to be important for stabilising the closed conformation of *Oa*IseP, in addition to triggering release of isethionate to the membrane subunits.

Another set of interactions considered important for the closure of TRAP substrate-binding proteins is the network of residues that co-ordinate the side chain of Arg156. Namely, a conserved histidine residue in the first spanning β-strand, as well as a conserved glutamate residue protruding from domain II [26,28]. In *Oa*IseP, the corresponding residue His239 is conserved, although the residue corresponding to glutamate is Gly216 (Figure 6D). In the isethionate-bound structure of *Oa*IseP, His239 is co-ordinated to the backbone carbonyl of Gly216, via two water molecules, which are also held by Glu184. The sidechain of Arg156 is held in place by a hydrogen bond to the backbone carbonyl of Gly216 on one side of the guanidyl group, as well as the sidechains of Asn240 and Ser242 on the opposing side. This is analogous to the conserved network in SiaP, where a glutamate residue in place of Gly216 is co-ordinated by the His239 equivalent to principally stabilise Arg156, with an asparagine residue hydrogen bonding the guanidyl group on the opposing side [28]. The residues at these positions have been suggested to be important for not only stabilising the closed conformation of TRAP substrate-binding proteins, but also for holding Arg156 in an orientation to accept the substrate in the open conformation. In the ligand-free structure of *Oa*IseP, two cryoprotectant molecules of ethylene glycol are present in the binding site, potentially perturbing the network of the open conformation and the position of Arg156 (Figure 6D). However, the rigid network formed in the closed conformation supports that these residues are key to stabilisation.

To assess whether the conformations of *Oa*IseP in our crystal structures were comparable in solution, we conducted small-angle X-ray scattering (SAXS) experiments in the presence and absence of isethionate. Scattering data are presented in Figure 6E and Supplementary Figure S4, with theoretical scattering curves calculated from the crystal structures fitted to the data using *CRYSOL* [45,46]. The calculated scattering profile of the ligand-free *Oa*IseP crystal structure (*χ*^2^ = 1.71) fits better than that of the scattering profile of the isethionate-bound *Oa*IseP crystal structure (*χ*^2^ = 2.31) to the experimental scattering data of *Oa*IseP in the absence of isethionate (Figure 6E, left pane). In contrast, the isethionate-bound *Oa*IseP structure (*χ*^2^ = 1.47) fits marginally better than the ligand-free *Oa*IseP structure (*χ*^2^ = 1.66) to the experimental scattering data of *Oa*IseP in the presence of 10 mM isethionate (Figure 6E, middle pane). A subtle decrease in the radius of gyration (*R_g_*) and maximum interatomic distance (*D*_max_) were also observed between *Oa*IseP in the absence of isethionate (*R_g_* = 20.8 Å, *D*_max_ = 65 Å) compared with *Oa*IseP in the presence of isethionate (*R_g_* = 20.5 Å, *D*_max_ = 63 Å) (Table 3), consistent with a slightly more compact structure from domain closure. These results indicate the structure of *Oa*IseP in solution resembles the crystal structures presented, supporting the conformational change occurring upon isethionate binding.

**Table 3.** Summary of structural parameters obtained for *Oa*IseP from SAXS

**SAXS data collection parameters**		
Instrument	Australian Synchrotron BioSAXS beamline	
Detector	Pilatus3X 2M (Dectris)	
Wavelength (Å)	1.002	
Maximum flux at sample	1 × 10^14^ photons per second at 12.0 keV	
Camera length (mm)	2500	
*q*-range (Å^−1^)	0.006–0.5	
Exposure time	Continuous 1-s frame measurements	
Sample configuration	SEC-SAXS with co-flow	
Sample temperature (°C)	20	
**SAXS data analysis**	**Ligand-free**	**Isethionate (10 mM)**
Guinier analysis		
*I_o_* (cm^−1^)	0.007 (±1.2) × 10^−5^	0.007 (±1.2) × 10^−5^
*R_g_* (Å)	20.8	20.5
*P*(*r*) analysis		
*I_o_* (cm^−1^)	0.01	0.01
*R_g_* (Å)	20.8	20.3
*D*_max_ (Å)	65	63
Porod volume (Å^−3^)	55 597	53 547
CRYSOL analysis		
Open (*χ*^2^ value)	1.71	1.66
Closed (*χ*^2^ value)	2.31	1.47

During the screening of *Oa*IseP crystals without isethionate, data from two crystals contained significant electron density within the binding site. We had suspected *Oa*IseP had tightly bound a non-cognate ligand present during expression in *E. coli*, a phenomenon that is not uncommon for TRAP substrate-binding proteins as documented by Vetting et al. [6]. Upon further refinement, we were able to confidently model HEPES and MES buffer constituents from the crystallisation conditions into the densities (difference maps shown in Supplementary Figure S5) and refined the structures to an *R*_free_ of 0.229 (1.89 Å resolution) and 0.241 (1.65 Å resolution) respectively (Table 2). A structural alignment of the four *Oa*IseP structures is presented in Figure 6F, demonstrating the HEPES- and MES-bound structures adopt the open conformation (the r.m.s.d. between the isethionate-, HEPES-, and MES-bound structures with the ligand-free structure over all 308 pairs of α-carbons are 2.7, 0.57, and 0.23 Å respectively). A closer examination of the binding sites in Figure 6G,H reveals that the sulfonate moiety of HEPES and MES forms similar contacts to *Oa*IseP, compared with the sulfonate of isethionate. In both HEPES- and MES-bound structures, the sulfonate groups form a salt bridge to Arg177 and hydrogen bonding the side chain nitrogen of Asn217. In the HEPES-bound structure (Figure 6G), Arg156 is tilted slightly further from the sulfonate group, only granting a single nitrogen atom of the guanidyl group to form hydrogen bonds with the HEPES sulfonate, whereas Arg156 is turned slightly closer to the MES sulfonate allowing the respective salt bridge to form (Figure 6H).

In the binding site opposite the sulfonate group, HEPES forms a hydrogen bond with its terminal hydroxyl group to the backbone nitrogen of Ala40 (Figure 6G). The smaller MES utilises a bridging water molecule hydrogen bond Ala40 to the morpholine ring oxygen (Figure 6H). HEPES also forms another hydrogen bond to a water molecule, above the piperazine ring via the nitrogen atom. This water molecule then forms a hydrogen bond with Tyr153, but in the MES-bound structure, no interaction is present. No other water molecules present in the binding site co-ordinate the ligands in either structure. While HEPES fills the binding site, making contacts with both domains I and II, it appears that closure is precluded by steric hindrance in the current orientation. In contrast, it is interesting that the slightly smaller MES does not induce partial closure, considering the water molecule bridging the hydrogen bond with Ala40 could be removed and a direct hydrogen bond could be formed instead. The melting temperature of *Oa*IseP ($T_{m}^{D}$) in the presence of MES (5 mM, 58.5 ± 0.3°C) and HEPES (5 mM, 57.0 ± 0.6°C) is not significantly different to that of ligand-free *Oa*IseP ($\Delta T_{m}^{D}$ of 0.6°C and −0.9°C, respectively). This suggests that these molecules bind weakly, or that it is the full closure of the protein around the ligand that induces thermal stability.

## Discussion

We set out to define the mechanism of isethionate import from the bacterium *O. alaskensis*, which is catalysed by the TRAP transporter *Oa*IsePQM. The utilisation of isethionate as an energy source in *Desulfovibrio* species generates hydrogen sulfide, which has implications for human health and potential industrial applications. The results reported here inform the mechanism of isethionate binding to *Oa*IseP and suggest why this protein is highly specific for isethionate.

### Mechanism of isethionate binding

The X-ray structures of *Oa*IseP with and without isethionate suggest why isethionate binds with high affinity. From our characterisation of *Oa*IseP, we predict that the mechanism of binding can be considered a two-step process: first, isethionate binds to the substrate-binding site and then triggers closure of two domains around the substrate.

(1) In the first step, the sulfonate group of isethionate forms two salt bridges with Arg156 and Arg177, and a hydrogen bond with Asn217 all located in domain II of *Oa*IseP. These interactions are likely the initial binding interaction because they are probably the strongest interactions between the isethionate and the protein. A similar initial step has been suggested for other TRAP substrate-binding proteins [18,26–28].
(2) In the second step, the formation of hydrogen bonds between the side chains of the hinge residues Ser242, Arg156, and Tyr153 triggers closure of the domains around isethionate. The closure of the domains allows for the domain I residues to come into proximity of isethionate and form the remaining hydrogen bonds with Ser95, His244, Asp77 and Asn98 (Figure 5D). There are also extensive interdomain interactions formed upon closure around isethionate, including the latch region that comprises residues, Asp77, Arg78, Asn98, Asn101, Asn180 (Figure 6C).

Given the small size of the substrate it is interesting that no water molecules are directly involved in coordinating isethionate. In comparison, larger TRAP transporter substrates such as sialic acid utilise water mediated interactions to SiaP [26]. There is, however, a water network around the latch residues that together stabilise the closed conformation. For both *Oa*IseP and SiaP, the water networks are involved in bridging the domains in the closed conformation. The culmination of interactions with isethionate in addition to the interdomain interactions stabilise the closed conformation (as seen in Figure 6C). Presumably, when in complex with the *Oa*IseQM membrane transporter component, interactions with the latch residues and or disruption of the water network would destabilise the closed conformation of *Oa*IseP and allow opening for isethionate transport across the membrane.

### Specificity

TRAP substrate-binding proteins are known to be highly specific for their substrate and binding is often in the nM range [28,39–41,43,47]. Given the diversity of phosphate- or sulfate-containing molecules found in biology, we were curious about whether *Oa*IseP was similarly specific and how specificity might be afforded. To address the first point, we screened a panel of biologically relevant phosphate or sulfate-containing molecules and found that only isethionate within this panel significantly stabilised *Oa*IseP. This corroborates the assertion that isethionate is the biological substrate of the *Oa*IsePQM TRAP transporter, but also suggests that *Oa*IseP is highly specific for isethionate. Further experiments demonstrate that *Oa*IseP binds isethionate with nM affinity (950 nM). Of note, similar sized molecules with a sulfonate moiety failed to induce thermal stabilisation. These include taurine, which has an amine group in the hydroxyl position of isethionate, butane-sulfonate, which has a four-carbon chain and no hydroxyl, and methyl-sulfonate, which has a single-carbon chain and no hydroxyl (Supplementary Table S3, red border). This indicates that the presence of the hydroxyl group is important for stabilising the closed conformation.

Serendipitously, we solved two crystal structures with sulfonate-containing buffers, HEPES and MES, within the substrate-binding site of *Oa*IseP. However, they do not trigger closure of the domains because they are either too large or do not adequately bridge the two domains. In comparison with isethionate, HEPES and MES only hydrogen bond Arg156, Arg177, Asn217, Ala40 (via a water molecule in MES) and Tyr153 (only present in HEPES via a water molecule), whereas isethionate additionally binds Ser95, Asp77, Asn98 and His244 (Figures 5D and 6G,H). Since the sulfonate moiety of all three ligands binds *Oa*IseP similarly (interacting with domain II residues Arg156, Arg177 and Asn217), we infer that the additional bridging interactions from isethionate to the two domains must stabilise the closed conformation. The larger size of HEPES and MES likely restrict the ability of the domains to close and form stabilising interactions, including those of the hinge residues Tyr153 and Ser242 with the sulfonate group and Arg156, and the interdomain interaction, including the water network.

In comparison with the TRAP substrate-binding proteins with native phosphate-containing substrates, *Oa*IseP differs in the way it co-ordinates the sulfonate group of isethionate (Figure 5G–I). The protein-substrate interactions in the phosphate-bound structures suggest that the lysine residues play a crucial role in differentiating carboxylates and phosphate-containing ligands. In the diglycerol-phosphate-bound structure, selection against glycerol phosphate is achieved with the lack of an analogous lysine and introduction of a tyrosine residue at a nearby position. *Oa*IseP does not contain a lysine or tyrosine at either position (neither do SiaP) but still forms a comprehensive network with the sulfonate moiety of isethionate. Hence, the additional hydrogen bonds between the sulfonate group and Ser95, Asn98, and Tyr153 create a unique binding site organisation for sulfonate groups.

**Figure 7.**
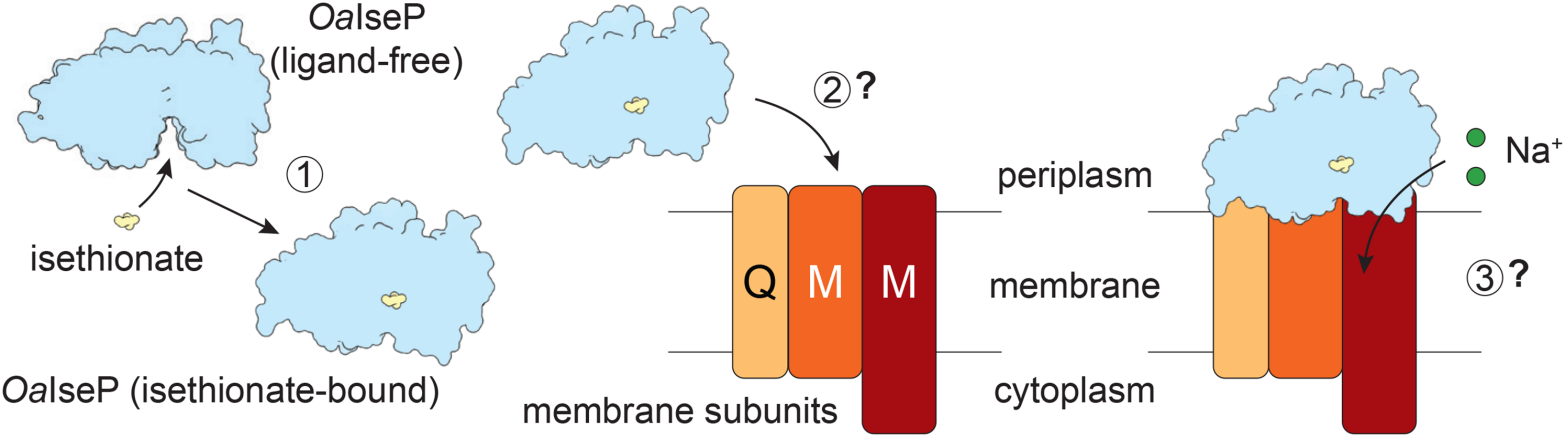
Schematic of the first steps in isethionate transport. This research has characterised the first step of transport, whereby *Oa*IseP in the open ligand-free conformation specifically binds isethionate, resulting in a conformational change to the closed form (1). *Oa*IseP must then deliver the isethionate to the membrane subunits (2) for transport through the membrane (3). The interactions between *Oa*IseP and the membrane subunits have not yet been determined and require further investigation.

To conclude, we present functional and structural data of the first sulfonate-specific TRAP substrate-binding protein, *Oa*IseP. Our investigation helps understand the initial stages of the TRAP transporter mechanism (Figure 7), detailing how *Oa*IseP selects for and binds isethionate. The next key knowledge gap is understanding how *Oa*IseP delivers isethionate to the membrane subunits for transport into the cell. Structural determination of the membrane subunits, as well as a model of how they interact with *Oa*IseP, will significantly aid our understanding of the mechanisms of sulfonate import in *Desulfovibrio*. Knowledge of the interactions formed between *Oa*IseP and isethionate could be leveraged to inform future protein engineering efforts using TRAP substrate-binding proteins. Finally, with the growing clinical relevance of *Desulfovibrio*, our work will help inform antibiotic development targeting sulfonate transport as a means of inhibiting hydrogen sulfide production within the human gut.

## Experimental procedures

### Multiple sequence alignment

A Position-Specific Iterated protein (PSI-)BLAST was performed using the *O. alaskensis* IseP protein sequence (NCBI accession no. WP_011367274, UniProt ID: Q312S0) as the query to identify homologous substrate-binding proteins. The search was conducted with the sequences of structures deposited in the PDB, and the top 10 highest scoring were chosen for sequence alignment and structural comparison. The multiple sequence alignment was performed using *Clustal Omega* [48] and the figure was produced in *ESPript* v3.0 [49].

### Protein expression and purification

The *O. alaskensis iseP* gene, without the native signal peptide, was synthesised by GENEWIZ and cloned into a modified pET21a(+) expression vector (containing an N-terminal hexahistidine-tag and HRV 3C protease cleavage site). The vector was transformed into *E. coli* BL21 (DE3) cells. Cells were grown at 37°C with shaking at 180 rpm to an OD_600_ of 0.6–0.8 in Luria broth. Recombinant protein expression was induced with 1 mM isopropyl β-d-1-thiogalactopyranoside for 16 h at 26°C. Cells were collected by centrifugation at 8000***g*** for 5 min at 4°C, before resuspension in phosphate-buffered saline (PBS) pH 7.4, 0.5 mg/ml lysozyme and 0.1 mM phenylmethylsulfonyl fluoride (PMSF). The resuspended cells were lysed by ultrasonication at 70% amplitude in 0.5 s on 0.5 s off cycles for 10 min, using a Hielscher UP200S Ultrasonic Processor. Insoluble material was removed by centrifugation at 30 000***g*** at 4°C for 30 min. The clarified lysate was applied to a 5 ml HisTrap HP column (Cytiva) equilibrated in PBS pH 7.4 with 20 mM imidazole. The column was washed with 20 column volumes of equilibration buffer before the bound protein was eluted by equilibration buffer supplemented with 500 mM imidazole. Fractions containing *Oa*IseP (determined by SDS–PAGE analysis) were pooled and concentrated using 10 kDa molecular mass cut-off concentrators (Pall). For removal of the N-terminal purification tag, *Oa*IseP was incubated with HRV 3C protease in a 50:1 mass ratio (substrate to enzyme) at 4°C for 16 h, before being loaded onto a HiLoad 16/600 Superdex 200 size-exclusion column (Cytiva) equilibrated with 50 mM Tris pH 8.0, 150 mM NaCl. Fractions containing *Oa*IseP were pooled, concentrated and flash-frozen for storage at −80°C if not being used immediately. For subsequent experiments, the concentration of *Oa*IseP was determined using a Thermo Scientific NanoDrop One Microvolume UV-Vis Spectrophotometer, with an extinction coefficient of 34 380 M^−1^ cm^−1^ [Abs 0.1% (= 1 g/l) = 0.924] and a molecular mass of 37.188 kDa. For his-tag cleaved *Oa*IseP, an extinction coefficient of 34 380 M^−1^ cm^−1^ [Abs 0.1% (= 1 g/l) = 0.977] and a molecular mass of 35.204 kDa was used. For the crystallisation of *Oa*IseP with isethionate, all purification buffers were supplemented with 5 mM isethionate.

### Native mass spectrometry

Native mass spectrometry was performed using a Waters SYNAPT XS mass spectrometer, equipped with a 32k quadrupole. Samples were exchanged into 200 mM ammonium acetate using Bio-Rad Micro Bio-Spin columns (final sample concentration of 20 µM), before infusion using gold-coated nanospray capillaries, prepared in-house, with an approximate orifice size of 1 µm. The instrument was operated in positive-ion mode and tuned for the analysis of native-like protein ions.

### DSF (thermal shift assays)

DSF experiments were conducted using a QuantStudio 3 Real-Time PCR System (Applied Biosystems/Thermo Fisher Scientific). For experiments using the Phenotype MicroArray PM4A Biolog screen, the compounds were dissolved in 50 µl of 50 mM Tris pH 8.0, 150 mM NaCl buffer, before 7.5 µl was used in each replicate (Biolog does not disclose the exact concentrations in their screens).

Each well was loaded with 25 µl of conditions comprising 15 µM *Oa*IseP in 50 mM Tris pH 8.0, 150 mM NaCl together with 10X SYPRO Orange dye (Invitrogen) and either 0.1 or 5 mM isethionate. The temperature was gradually increased from 4°C to 95°C at a rate of 0.03°C/s and the fluorescence intensity was measured over time. *Protein Thermal Shift Software* v1.4 (Applied Biosystems) was used to analyse the data using the derivative melting temperature function.

### ITC (binding affinity)

ITC experiments were conducted using a TA Instruments Low Volume NanoITC. *Oa*IseP was diluted to a concentration of 100 µM in 50 mM Tris pH 8.0, 150 mM NaCl, and loaded into the sample cell. The injection syringe was filled with 1 mM isethionate in the same buffer, while the reference cell was filled with water. The system was allowed to equilibrate with stirring at 400 rpm, before an initial 1 µl injection was started, followed by 24 injections of 2 µl at 180 s intervals. *SEDPHAT* [50] was used to analyse the triplicate data by a global fit, excluding the first points of each dataset.

### Analytical ultracentrifugation

Sedimentation velocity AUC was performed using a Beckman Coulter Optima analytical ultracentrifuge, with a Beckman Coulter AN50Ti rotor with quartz windowed cells. *Oa*IseP at 0.1 and 0.9 mg/ml (2.8 and 25.6 μM) was analysed in 50 mM Tris pH 8.0, 150 mM NaCl in the absence of isethionate and the presence of 5 mM isethionate. Assays were performed at 20°C with absorbance optics at 226 nm (0.1 mg/ml) and 241 nm (0.9 mg/ml) and run at 50 000 rpm. Sedimentation data were analysed with *UltraScan* v4.0 [51]. Optimisation was performed by two-dimensional spectrum analysis (2DSA) [52] with simultaneous removal of time- and radially-invariant noise contributions and fitting of boundary conditions. Processing was done using a 64 × 64 resolution grid with the bounds of 1–4 for the frictional ratio and 1–10 for the sedimentation coefficient. Following iterative fitting, the 2DSA solutions were subjected to parsimonious regularisation by genetic algorithm analysis [53]. The statistics of the final fitted model are reported in Supplementary Table S4.

### Crystallisation and structure determination

Crystallisation trials were set up using sitting drop vapour diffusion with JCSG-plus, PACT premier and SG1 screens (Molecular Dimensions). Drops consisting of 400 nl of condition and 400 µl of protein solution (*Oa*IseP at 25–50 mg/ml with and without 5 mM isethionate in 50 mM Tris pH 8.0, 150 mM NaCl) were mixed using the Mosquito Protein Crystallization System (SPT Labtech) and incubated at 20°C. Crystals that grew in; PACT premier G2 (0.2 M sodium bromide, 0.1 M Bis-Tris propane pH 7.5, 20% w/v PEG 3350), PACT premier E5 (0.2 M sodium nitrate, 20% w/v PEG 3350), PACT premier B9 (0.2 M lithium chloride, 0.1 M MES pH 6.0, 20% w/v PEG 6000) and SG1 A6 (0.1 M HEPES pH 7.5, 20% PEG 4000, 10% 2-propanol), for ligand-free, isethionate-bound, MES-bound and HEPES-bound, respectively, were selected for data collection. Crystals were mounted in loops and preserved in 15% (v/v) cryoprotectant (50% (v/v) ethylene glycol, 50% (v/v) glycerol), before being cooled in liquid nitrogen prior to data collection.

Diffraction data for *Oa*IseP were collected at the Australian Synchrotron (Melbourne, Australia) on the MX2 beamline using an X-ray wavelength of 0.954 Å equipped with an EIGER 16M detector [54]. Diffraction data were scaled and processed using *XDS* and *AIMLESS* in the *CCP4i2* suite [55]. Resolution cutoffs were determined following the criteria that the CC_1/2_ of each dataset is above 0.35, the *I*/*σI* was equal to or greater than 1.0 and completeness was above 95%. Data collection statistics are reported in Table 2.

Structures were determined by molecular replacement using *MOLREP* [56], part of the *CCP4i2* suite [55]. Initially, ligand-free *Oa*IseP was solved using a *CHAINSAW*[57] prepared search model derived from the structure of ligand-free *Hi*SiaP (PDB ID: 2CEY). Subsequent structures were then solved using the refined ligand-free *Oa*IseP structure. For all structures, initial manual model rebuilding was carried out in *Coot* and rounds of refinement were conducted using *REFMAC5* [58]. Water molecules were identified and added in the later stages of refinement. Refinement statistics are reported in Table 2.

### Small-angle X-ray scattering

Purified *Oa*IseP was exchanged into PBS pH 7.4 with or without 10 mM isethionate before SAXS data collection. Data were collected at the Australian Synchrotron (Melbourne, Australia) on the BioSAXS beamline equipped with a Pilatus3X 2M detector (pixel size 172 × 172 µm). A sample detector distance of 2500 mm was used, providing a *q* range of 0.006–0.5 Å^−1^. Here, 60 µl of purified *Oa*IseP at 3 mg/ml was injected onto an inline Superdex 200 Increase 5/150 GL SEC column (Cytiva), equilibrated with PBS pH 7.4, supplemented with 2% glycerol, using a flow rate of 0.3 ml/min. For data collected in the presence of isethionate, the same buffer was used with isethionate added to a final concentration of 10 mM. Scattering data were collected in one second exposures (*λ* = 1.002 Å) over a total of 600 frames, using a 1.5 mm glass capillary, at 20°C. Data calibration and reduction was performed using customised algorithms written for the BioSAXS beamline at the Australian Synchrotron. The scattering vector (*q*) was calibrated using calibration standards with known scattering peaks. Scattering patterns were reduced using the pyFAI-saxs library for Fast Azimuthal Integration. Reduced data was processed and further analysed using CHROMIXS in the *ATSAS v3.2.1* suite [59]. The theoretical scattering profile, *R_g_* and *D*_max_ values for the open, and closed isethionate-bound conformations were calculated in *CRYSOL* [45,60] and compared with the experimental data. The parameters for data collection and processing are summarised in Table 3.

### Software

Figures were created using *UCSF ChimeraX* v1.8 [61], *GraphPad Prism* v10 and *Adobe Illustrator* 2024.

## Data Availability

All data will be made available on request to the corresponding author. X-ray crystal structures and data are accessible through the Protein Data Bank [PDB IDs: 8T9T (ligand-free), 8TE9 (isethionate-bound), 8TRP (HEPES-bound) and 8TQN (MES-bound)] [62–65].

## Open Access

Open access for this article was enabled by the participation of University of Canterbury in an all-inclusive *Read & Publish* agreement with Portland Press and the Biochemical Society under a transformative agreement with CAUL.

## Abbreviations

AUC: analytical ultracentrifugation
DSF: differential scanning fluorimetry
ITC: isothermal titration calorimetry
PBS: phosphate-buffered saline
SAXS: small-angle X-ray scattering
smFRET: single-molecule Förster Resonance Energy Transfer
