# Supplementary material for: On the function of TRAP substrate-binding proteins: the isethionate-specific binding protein IseP
Source: Biochem J. 2024 Dec 9;481(24):1901–20. doi: 10.1042/BCJ20240540 (PMC11668362; doi:10.1042/BCJ20240540)
Supplement: Supplementary Material 1 [file BCJ-481-1901-s1.pdf]

Supplementary Information, Tables, and Figures for:

On the function of TRAP substrate-binding proteins: the isethionate-specific binding protein IseP

Michael C. Newton-Vesty, Michael J. Currie, James S. Davies, Santosh Panjikar, Ashish Sethi, Andrew E. Whitten, Zachary D. Tillett, David M. Wood, Joshua D. Wright, Michael J. Love, Timothy M. Allison, Sam A. Jamieson, Peter D. Mace, Rachel A. North & Renwick C.J. Dobson

Supplementary Tables

Table S1 | Results from the PSI-BLAST similarity search of the *Oa*IseP amino acid sequence (NCBI accession no. WP_011367274, UniProt ID: Q312S0), limited to structures deposited in the PDB.

PDB ID	Gene	Ligand	Species
4P47	*oant_4429*	C-terminus (open)	*Brucella anthropi* ATCC 49188
4P9K	*veis_3954*	pantoate or erythronate	*Verminephrobacter eiseniae* EF01-2
4NQ8	*bb3421*	pantoate	*Bordetella bronchiseptica* RB50
4PDH	*bpro_1871*	erythronate	*Polaromonas sp.* JS666
7BBR	*cxaP/dctP*$_{Am}$	2-keto-3-deoxygluconate	*Advenella mimigardefordensis* DPN7^{T}
4NN3	*desal_2161*	orotic acid	*Maridesulfovibrio salexigens* DSM 2638
4N8Y	*bbta_0128*	galacturonate	*Bradyrhizobium sp.* BTAi1
4X8R	*rsph17029_2138*	glucuronate	*Cereibacter sphaeroides* ATCC 17029
4XEQ	*deval_0042*	pantoate	*Nitratidesulfovibrio vulgaris* RCH1
4PFR	*rsph17029_3541*	malate (open)	*Cereibacter sphaeroides* ATCC 17029

Table S2 | Protein sequences of *Oa*IseP used in this work. The signal peptide sequence predicted by SignalP 6.0 [1] is underlined and the N-terminal his-tag and HRV 3C protease cleavage site are coloured.

**Native** (NCBI accession no. WP_011367274, UniProt ID: Q312S0)	<u>MKHLLKAGALVALACIVTLTAGAQAHA</u>AKRINIRLAHPMAPGNNVTVG<br>YEKFKELVAEKSNGRVRIQLFGNCMLGSDRVTMEAAQRGTLEMASSSS<br>PNMANFSKQWMVFDLPYITSPEHQQKLYKAIDDGELGKKLDEIAASIGL<br>KPIMYSEYGYRNFVTTKKPIKTADDLKNLKVRTTDSPIEVAVAAALGMA<br>PTPISWGETYTALQQGTVDGEGNTFSLLNDAKHTEVLKYAIDSAHNYSM<br>HLLMMNKAYYDSLPANVQQILTEAGREALTYQRSITSELEKKAEDAFIE<br>QGITVTRLSPEERAKLVERTRPVWDKFKDDIPAELIKLVQETQQ
**Expression**	MAHHHHHHSAALEVLFQGPGQAHAAKRINIRLAHPMAPGNNVTVGYE<br>KFKELVAEKSNGRVRIQLFGNCMLGSDRVTMEAAQRGTLEMASSSSPN<br>MANFSKQWMVFDLPYITSPEHQQKLYKAIDDGELGKKLDEIAASIGLKPI<br>MYSEYGYRNFVTTKKPIKTADDLKNLKVRTTDSPIEVAVAAALGMAPTP<br>ISWGETYTALQQGTVDGEGNTFSLLNDAKHTEVLKYAIDSAHNYSMHL<br>LMMNKAYYDSLPANVQQILTEAGREALTYQRSITSELEKKAEDAFIEQ<br>GITVTRLSPEERAKLVERTR PVWDKFKDDIPAELIKLVQETQQ
**His-tag cleaved**	GPGQAHAAKRINIRLAHPMAPGNNVTVGYEKFKELVAEKSNGRVRIQLF<br>GNCMLGSDRVTMEAAQRGTLEMASSSSPNMANFSKQWMVFDLPYITSP<br>EHQQKLYKAIDDGELGKKLDEIAASIGLKPIMYSEYGYRNFVTTKKPIKT<br>ADDLKNLKVRTTDSPIEVAVAAALGMAPTPISWGETYTALQQGTVDGE<br>GNTFSLLNDAKHTEVLKYAIDSAHNYSMHLLMMNKAYYDSLPANVQQI<br>LTEAGREALTYQRSITSELEKKAEDAFIEQGITVTRLSPEERAKLVERTRP<br>VWDKFKDDIPAELIKLVQETQQ

Table S3 | Tabulated ΔT_m^D for the DSF experiments using the Phenotype MicroArray PM4A screen. These data are as shown in the thermal shift heatmap in **Figure 3A**. The mean and standard deviation from technical quadruplicates of each condition are tabulated. Isethionate (2-hydroxyethane sulfonic acid, position H10, purple) was the only hit (ΔT_m^D > 2 °C) amongst 94 other metabolites from the screen. Compounds with significant structural similarity to isethionate are indicated with a red border (taurine, butane-sulfonate, and methyl-sulfonate).

	ΔT_m^D (°C)							
	A	B	C	D	E	F	G	H
1	negative control 0.00 ± 0.15	thiophosphate -0.04 ± 0.31	phosphoenolpyruvate -0.63 ± 0.24	D-mannose-1-phosphate 0.69 ± 0.31	O-phospho-D-tyrosine 0.87 ± 0.11	negative control 0.00 ± 0.24	N-acetyl-L-cysteine -0.64 ± 0.24	L-djenkolic acid -0.32 ± 0.28
2	phosphate -0.09 ± 0.54	dithiophosphate 0.14 ± 0.22	phospho-glycolic acid 0.31 ± 0.21	D-mannose-6-phosphate -0.09 ± 0.47	O-phospho-L-tyrosine -0.73 ± 0.33	sulfate -0.37 ± 0.18	S-methyl-L-cysteine -0.41 ± 0.48	thiourea -0.73 ± 0.31
3	pyrophosphate 1.10 ± 0.58	DL-a-glycerol phosphate 1.28 ± 0.18	D-glucose-1-phosphate 0.92 ± 0.26	cysteamine-S-phosphate 0.27 ± 0.32	phospho-creatine 0.41 ± 0.40	thiosulfate 1.64 ± 0.25	cystathionine 0.61 ± 0.21	1-thio-b-D-glucose 0.45 ± 0.13
4	trimetaphosphate -0.73 ± 0.18	b-glycerol phosphate -0.30 ± 0.29	D-glucose-6-phosphate -0.85 ± 0.37	phospho-L-arginine -0.73 ± 0.17	phosphoryl-choline 0.05 ± 0.09	tetrathionate 1.01 ± 0.10	lanthionine -0.46 ± 0.10	DL-lipoamide 0.60 ± 0.38
5	tripolyphosphate -0.18 ± 0.31	carbamoyl phosphate -0.49 ± 0.28	2-deoxy-D-glucose 6-phosphate -0.19 ± 0.18	O-phospho-D-serine 0.05 ± 0.42	O-phosphoryl-ethanolamine -0.24 ± 0.10	thiophosphate 0.64 ± 0.19	glutathione -0.67 ± 0.37	taurocholic acid -1.46 ± 0.36
6	triethyl phosphate -0.19 ± 0.00	D-2-phospho-glyceric acid 0.12 ± 0.39	D-glucosamine-6-phosphate 0.31 ± 0.22	O-phospho-L-serine -0.37 ± 0.00	phosphonoacetic acid 0.24 ± 0.22	dithiophosphate 0.35 ± 0.00	DL-ethionine 0.14 ± 0.38	taurine 0.11 ± 0.46
7	hypophosphite -0.92 ± 0.18	D-3-phospho-glyceric acid -0.86 ± 0.21	6-phospho-gluconic acid -0.62 ± 0.42	O-phospho-L-threonine -0.62 ± 0.28	2-aminoethyl phosphonic acid -0.56 ± 0.37	L-cysteine -0.68 ± 0.11	L-methionine -0.92 ± 0.37	hypotaurine -0.68 ± 0.46
8	adenosine 2`-monophosphate -0.19 ± 0.32	guanosine 2` monophosphate -0.74 ± 0.00	cytidine 2`-monophosphate -0.65 ± 0.13	uridine 2`-monophosphate 0.24 ± 0.28	methylenediphosphonic acid 0.24 ± 0.38	D-cysteine 1.14 ± 0.17	D-methionine -0.62 ± 0.38	p-aminobenzene sulfonate -0.44 ± 0.38
9	adenosine 3`-monophosphate 0.18 ± 0.37	guanosine 3`-monophosphate -0.25 ± 0.28	cytidine 3`-monophosphate -0.65 ± 0.13	uridine 3`-monophosphate 0.24 ± 0.28	thymidine 3`-monophosphate -0.56 ± 0.37	Cys-Gly 1.14 ± 0.17	Gly-Met -0.01 ± 0.18	butane sulfonate 0.41 ± 0.46
10	adenosine 5`-monophosphate -0.38 ± 0.26	guanosine 5`-monophosphate -0.20 ± 0.32	cytidine 5`-monophosphate 0.17 ± 0.37	uridine 5`-monophosphate 0.17 ± 0.48	thymidine 5`-monophosphate 0.24 ± 0.38	L-cysteic acid 0.11 ± 0.28	N-acetyl-DL-methionine 0.23 ± 0.28	2-hydroxyethane sulfonic acid 6.52 ± 0.18
11	adenosine 2`,3`-cyclic monophosphate 0.14 ± 0.09	guanosine 2`,3`-cyclic monophosphate 0.91 ± 0.56	cytidine 2`,3`-cyclic monophosphate -0.07 ± 0.28	uridine 2`,3`-cyclic monophosphate 0.05 ± 0.11	inositol hexaphosphate 0.46 ± 0.33	cysteamine 0.35 ± 0.00	L-methionine sulfoxide 0.29 ± 0.11	methane sulfonic acid 0.23 ± 0.28
12	adenosine 3`,5`-cyclic monophosphate 0.23 ± 0.28	guanosine 2`,3`-cyclic monophosphate -0.50 ± 0.46	cytidine 3`,5`-cyclic monophosphate -0.38 ± 0.18	uridine 3`,5`-cyclic monophosphate 0.05 ± 0.21	thymidine 3`,5`-cyclic monophosphate 0.35 ± 0.18	L-cysteine sulfinate 0.53 ± 0.00	L-methionine sulfone 0.44 ± 0.13	tetramethylene sulfone 0.23 ± 0.11

Table S4 | Sedimentation velocity analysis of *Oa*IseP as plotted in **Figure 4**. Values were obtained using the *UltraScan* v4.0 software.

**Sample**	**Concentration mg/mL (µM)**	**Wavelength (nm)**	**Peak $S_{20,w}$ (S)**	***f/f_0***	**Obtained mass (kDa)**	**Mass from sequence (kDa)**	**Variance**	**r.m.s.d.**
***Oa*IseP**	0.1 (2.8)	226	2.96	1.24	33.4	35.2	$1.52e^{-05}$	0.00390
	0.9 (25.6)	241	2.91	1.27	33.6	35.2	$9.38e^{-06}$	0.00306
***Oa*IseP + 5 mM isethionate**	0.1 (2.8)	226	3.02	1.27	35.2	35.2	$1.26e^{-05}$	0.00354
	0.9 (25.6)	241	2.98	1.26	34.5	35.2	$5.36e^{-06}$	0.00231

Supplementary Figures

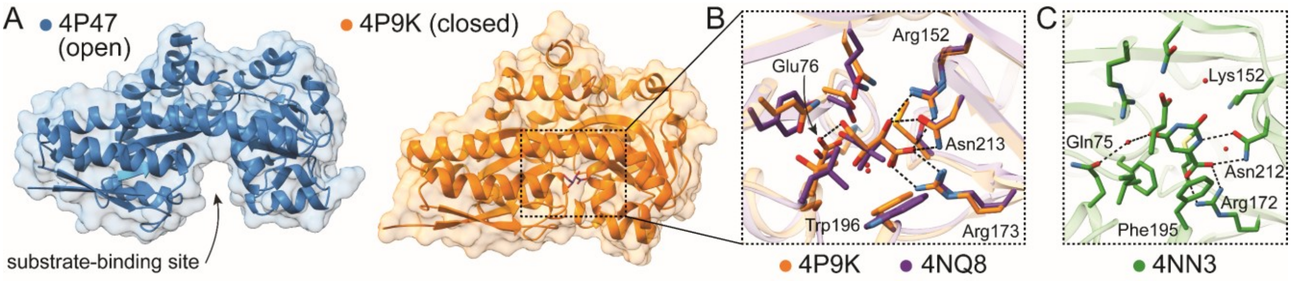

Figure S1 | Substrate-binding site analysis of *Oa*IseP homologues. A) The structure of 4P47 (blue) in the open conformation and 4P9K (orange) in the closed conformation with erythronate bound, representing the two conformations occupied by our sequence alignment hits. **B)** A structural overlay of two representative substrate-binding sites of 4P9K (gold), bound to erythronate, and 4NQ8 (purple), bound to pantoate. Residues are labelled according to the numbering of 4P47 for consistency with the multiple sequence alignment presented in **Figure 2**. The highly conserved residues are labelled, and the relevant hydrogen bonds are depicted in black; Arg173 forms a salt bridge with the carboxylate group of the ligand, while Arg152 and Asn213 provide supporting hydrogen bonds. The conserved aromatic residue (Trp196) provides a hydrophobic face for the carbon backbone of the ligand to rest against, while Glu76 commonly forms a hydrogen bond with a ligand hydroxyl group. **C)** The substrate-binding site of 4NN3 differs at highly conserved positions, with Lys152 (as opposed to arginine) facing away from the ligand carboxylate group, and Gln75 (as opposed to glutamate) involved in a hydrogen bond with the ligand *via* a water molecule.

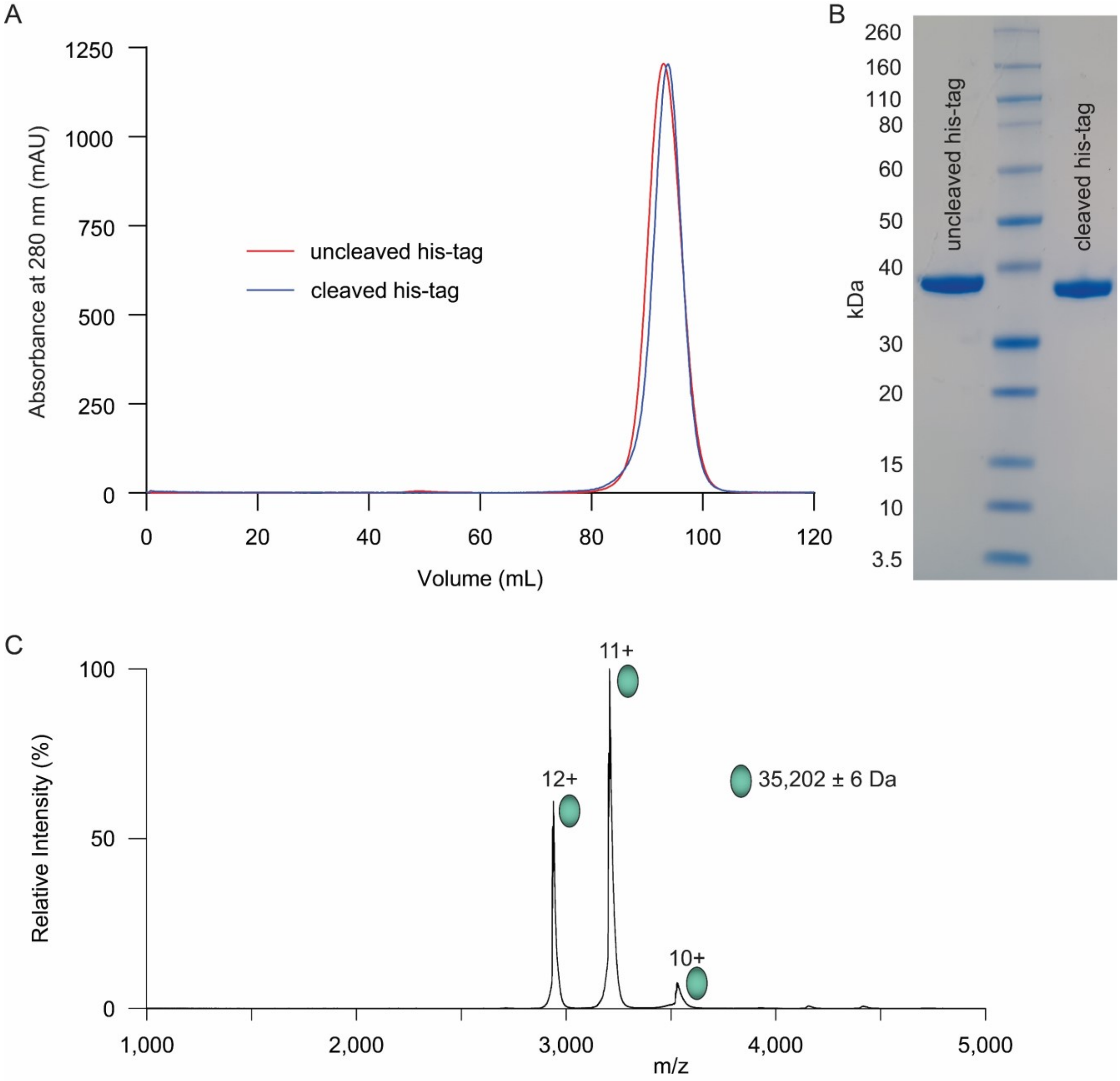

Figure S2 | *Oa*IseP purification. A) In the final purification step, *Oa*IseP was loaded onto a HiLoad 16/600 Superdex 200 size-exclusion column (Cytiva). Size-exclusion chromatograms of *Oa*IseP with (blue line) and without (red line) the N-terminal his-tag cleaved by HRV 3C protease are shown. **B)** SDS-PAGE analysis of *Oa*IseP, demonstrating >95% purity. **C)** Native mass spectrometry demonstrates that the mass of the protein is 35,202 ± 6 Da, consistent with the calculated mass from the protein sequence (35,204 Da) and a monomeric state.

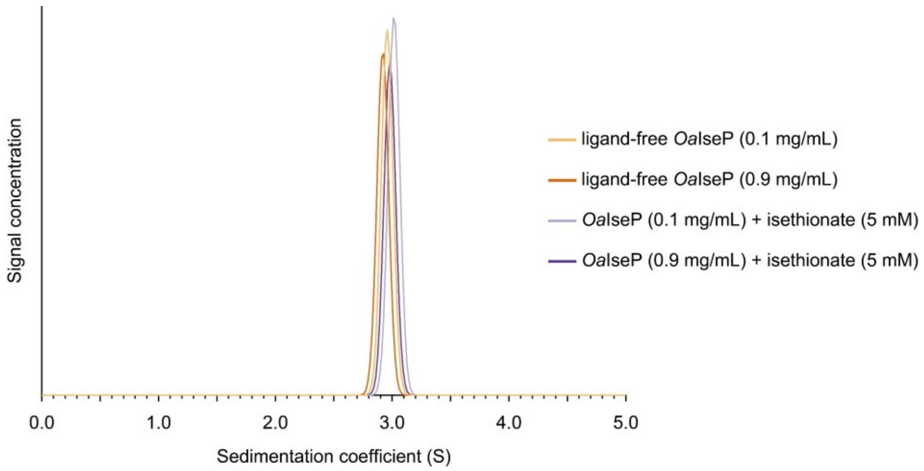

Figure S3 | *Oa*IseP sedimentation velocity analytical ultracentrifugation analysis. AUC sedimentation velocity profiles of *Oa*IseP, indicate that the protein is monomeric in solution with and without isethionate present.

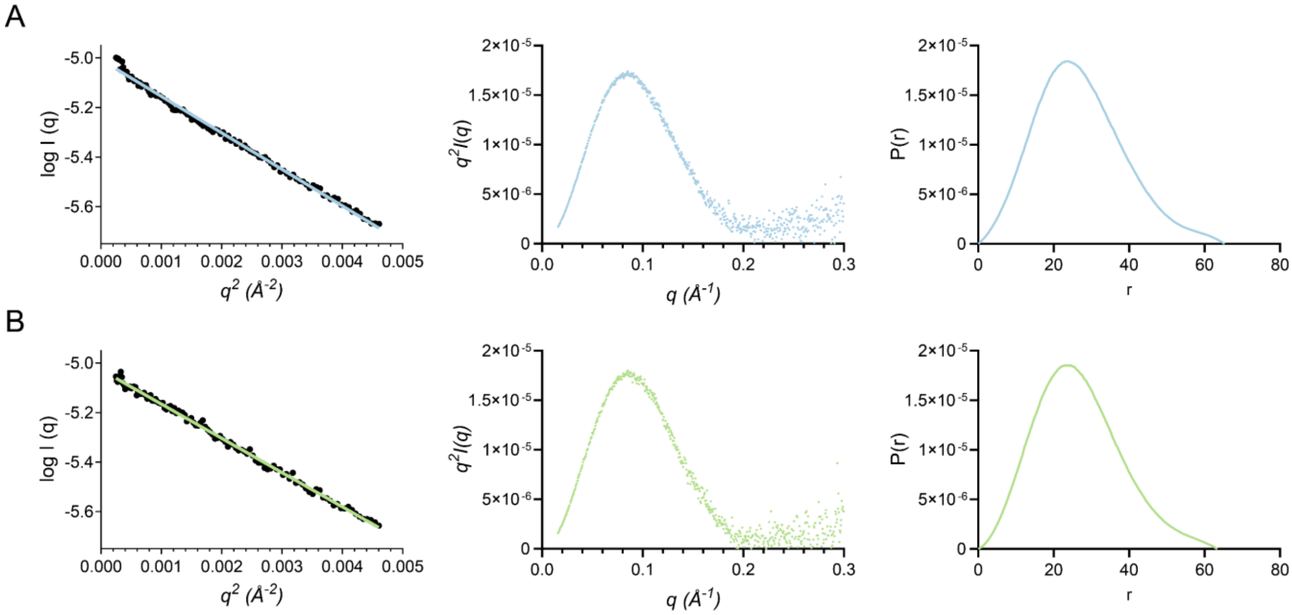

Figure S4 | *Oa*IseP small-angle X-ray scattering analysis. A) SAXS plots of *Oa*IseP without isethionate: Guinier, Kratky, and *P(r)*, from left to right. **B)** SAXS plots of *Oa*IseP with 10 mM isethionate: Guinier, Kratky, and *P(r)*, from left to right.

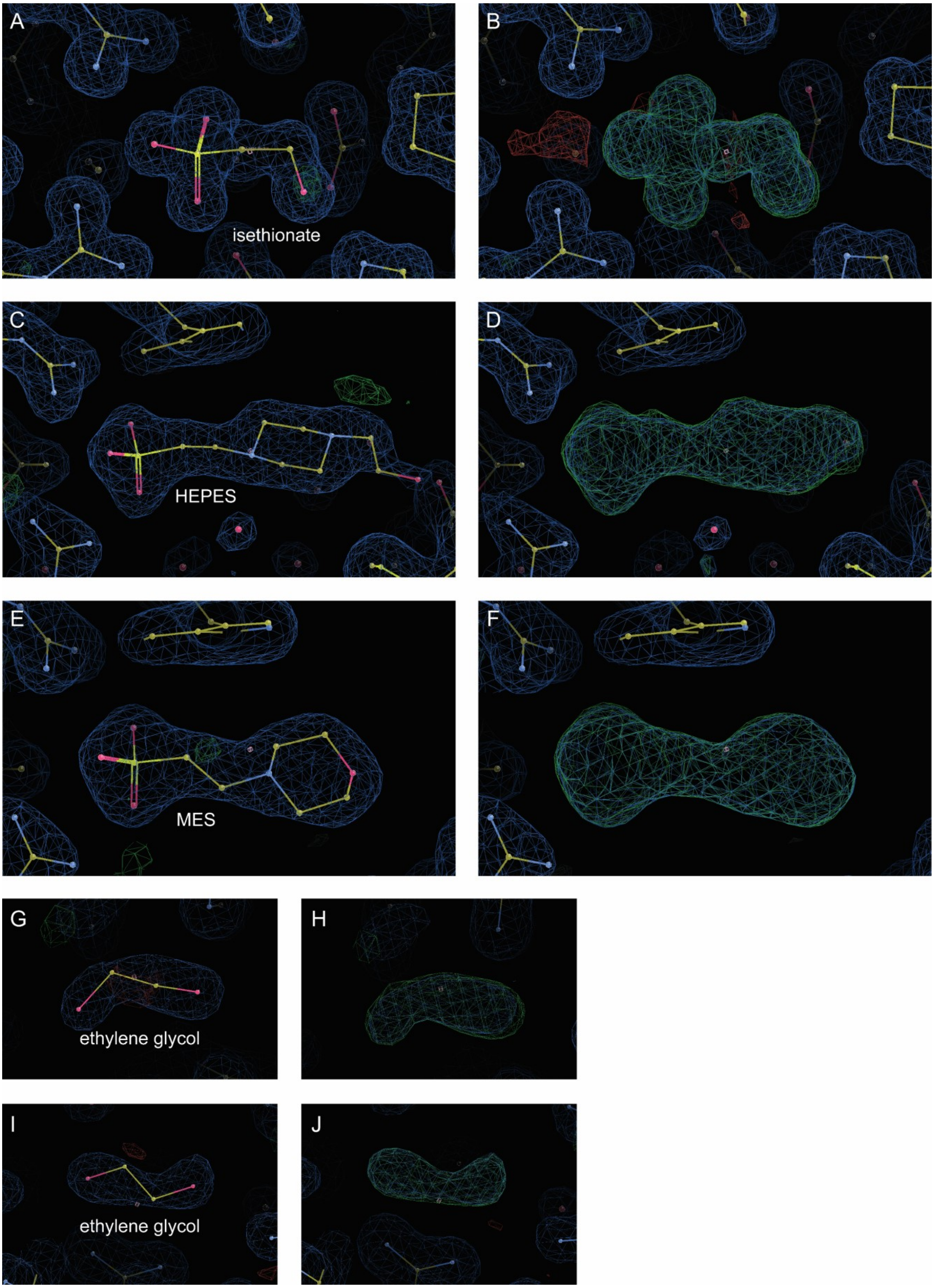

Figure S5 | Electron density maps of *Oa*IseP with ligands and omit maps. The *2Fo-Fc* maps (contoured to 1σ) are coloured blue, and the difference density *Fo-Fc* maps (contoured to +3σ green,

and -3σ red) for isethionate (**A and B**), HEPES (**C and D**), MES (**E and F**) and the ethylene glycol molecules present in the ligand-free structure of *Oa*IseP (**G-J**).

References

1. Teufel, F., Almagro Armenteros, J.J., Johansen, A.R., Gislason, M.H., Pihl, S.I., Tsirigos, K.D., et al. (2022) SignalP 6.0 predicts all five types of signal peptides using protein language models. Nat Biotechnol. **40**(7):1023-5. 10.1038/s41587-021-01156-3
